# Validation of Centers for Disease Control and Prevention level 3 risk classification for healthcare workers exposed to severe acute respiratory coronavirus virus 2 (SARS-CoV-2)

**DOI:** 10.1017/ice.2020.1353

**Published:** 2020-12-07

**Authors:** Candace M. Gragnani, Priyanka Fernandes, Daniel A. Waxman

**Affiliations:** 1 Department of Pediatrics, University of California–Los Angeles, Los Angeles, California; 2 Department of Medicine, University of California–Los Angeles, Los Angeles, California; 3 Department of Emergency Medicine, University of California–Los Angeles, Los Angeles, California

Experience from Wuhan, China, suggests that early identification and risk mitigation of healthcare workers (HCWs) potentially infected with coronavirus disease 2019 (COVID-19) is vital to preventing disease transmission in healthcare settings.^1^ Early on, the Centers for Disease Control and Prevention (CDC) recommended furloughing HCWs with medium- and high-risk workplace exposures to severe acute respiratory syndrome coronavirus 2 (SARS-CoV-2).^2^ They defined low-, medium-, and high-risk HCW exposures based on duration of close contact, presence of source control, and donning of personal protective equipment (PPE).^2^ We evaluated the performance of the CDC classification scheme when applied prospectively early in community transmission.

**Table 1.** Description of Healthcare Worker (HCW) Population with Centers for Disease Control and Prevention (CDC)–Risk Classified, Work-Related SARS-CoV-2 Exposures Enrolled in Symptom Monitoring (N=667)

Variable		CDC Exposure Risk Level, No. (% of Risk Level)		
	% Tested With PCR for SARS-CoV-2	High	Medium	Low
Population (N=667)	48.1	98	192	377
Age in y, Mean ± SD (N=576)		36.6±9.4	38.6±10.5	39.3±10.3
**Sex (N=572)**^a^				
Female (N=375)	53.3	53 (62.4)	110 (67.1)	212 (65.6)
Male (N=197)	41.6	32 (37.7)	54 (32.9)	111 (34.4)
**Employee job description**				
Nurse (N=274)	50.7	38 (38.8)	83 (43.2)	153 (40.6)
Resident physician (N=37)	67.6	5 (5.1)	12 (6.3)	20 (5.3)
Attending physician (N=47)	46.8	7 (7.1)	15 (7.8)	25 (6.6)
Respiratory therapist (N=28)	67.9	11 (11.2)	7 (3.7)	10 (2.7)
Other (clinical and support staff) (N=281)	41.3	37 (37.8)	75 (39.1)	169 (44.8)
**Employee job site**				
Inpatient (N=478)	50.8	82 (83.7)	140 (72.9)	256 (67.9)
Outpatient/other (N=189)	47.1	16 (16.3)	52 (27.1)	121 (32.1)
Days between exposure and enrollment, median [IQR] (N=667)		3 [2–6]	4 [3–6]	4 [2–7]
Days between enrollment and testing, median [IQR] (N=321)		3 [2–7]	4 [2–7]	4 [3–5]
Tested (N=321)		64 (65.3)	102 (53.1)	155 (41.1)
**Outcome**				
Positive COVID-19 PCR (N=24)^b^		9	9	6
As % of enrolled (95% CI)		9.2 (4.3–16.7)	4.7 (2.2–8.7)	1.6 (0.6–3.4)
As % of tested (95% CI)		14.1 (6.6–25.0)	8.8 (4.1–16.1)	3.9 (1.4–8.2)

NOTE. COVID-19, coronavirus disease 2019; SARS-CoV-2, severe acute respiratory coronavirus virus 2; PCR, polymerase chain reaction; SD, standard deviation; IQR, interquartile range; CI, confidence interval.

a % nonmissing.

b The Fisher exact tests for the association between risk category and a positive test in the exposed population and the tested population were *P* = .001 and *P* = .028, respectively.

## Methods

UCLA Health is a large academic center with 2 acute-care hospitals, a psychiatric hospital, and many ambulatory sites. The study population included all UCLA Health employees with healthcare-related exposures between March 9 and March 27, 2020. During this interval, 357 patients tested positive for SARS-CoV-2 across UCLA Health sites, and 1,465 cases of COVID-19 were documented in Los Angeles County.^3^ The infection prevention team was notified of all patients diagnosed with COVID-19 and was responsible for contact tracing, identifying potential employee exposure locations, and notifying unit supervisors and location managers at exposure locations. Supervisors and location managers would then (1) interview employees to confirm close contact (defined as <2 m for >3 minutes), (2) provide prospective CDC risk classification after an assessment of source control and employee PPE use, and (3) enroll them in a web-based symptom-tracking system. HCWs reporting symptoms were tested for SARS-CoV-2 at the physician’s discretion.

We matched records from the postexposure tracking system to a consolidated report of SARS-CoV-2 polymerase chain reaction (PCR) test results to identify tested HCWs. The primary study outcome was a positive test within 14 days of exposure identification and notification. Untested HCWs and those tested after 14 days were treated as nonpositive. For HCWs with multiple exposures, the first instance of the highest risk exposure was used. We calculated the proportion with a positive PCR along with 95% confidence intervals (binomial, exact), and we determined the significance of the association between risk category and a positive result using the Fisher exact test. We obtained review and approval with waiver of consent from our institutional review board.

## Results

In total, 753 HCWs were enrolled in postexposure monitoring. However, 45 HCWs were excluded from analysis because they had had SARS-CoV-2 testing between the date of exposure and enrollment, and 41 were excluded because the risk classification was missing. Population characteristics and outcomes are listed in Table 1. Of the 667 individuals included, exposure was classified as high risk for 98 (14.7%), medium risk for 192 (28.8%), and low risk for 377 (56.5%). Exposed HCWs were most commonly nurses (41.1%), and 71.7% of exposures occurred with inpatients (Table 1).

Overall, 321 HCWs (48.1%) were tested for SARS-CoV-2, and 24 (7.5%) were positive (Table 1). The Centers for Disease Control and Prevention (CDC) risk category was significantly associated with a positive test (*P* < .01). The proportions of HCWs with high-, medium-, and low-risk exposures diagnosed with COVID-19 were 9.2% (95% confidence interval [CI], 4.3%–16.7%), 4.7% (95% CI, 2.2%–8.7%), and 1.6% (95% CI, 0.6%–3.4%), respectively. This relationship remained significant (*P* = .03) when the analysis was restricted to the 321 HCWs tested.

## Discussion

The CDC’s 3-level risk classification was extrapolated from experience with other coronaviral infections including severe acute respiratory syndrome (SARS) and Middle East respiratory syndrome (MERS).^4,5^ In this study, we validated the CDC’s initial exposure risk model for COVID-19, and we quantified the probability of infection by risk classification when applied prospectively in a real-world setting.

The 9.2% infection rate we observed among HCWs with high-risk exposure is consistent with previously published data.^6,7^ However, our finding differs in demonstrating a full gradient of risk, with infection rates associated with medium-risk exposures intermediate between low- and high-risk groups. The observed “dose–response” between exposure severity and infection rates suggests a causal relationship during the study period.

This study has several limitations. Only 48% of the population was tested, and testing was more common after higher-risk exposures, which may have introduced bias. However, results were similar when only the subpopulation tested was considered. Also, although HCWs with high- and medium-risk exposures were initially furloughed, HCWs with low-risk exposures were allowed to continue working. This factor increased the risk of misattribution and may have biased the results toward the null hypothesis of no association. Finally, absolute infection rates represent an average across diverse facilities and might be different at specific facilities, according to environmental controls.

Notably, the CDC’s subsequently revised guidance provides a simplified 2-level classification scheme grouping medium-risk exposures (source control but no HCW facemask or respirator, or no source control and no HCW eye protection) together with high-risk exposures. The minimum duration of close contact that meets exposure definition was changed from a “few minutes” to 15 cumulative minutes in a 24-hour period.^2,8^ The importance of contact duration and distance in classifying exposure risk, in addition to the relative benefit of facemasks versus respirators and eye protection, remains unresolved.

Discrete, well-identified exposures to infected patients might not be the predominant risk to HCWs in the current high-prevalence environment, where HCW-to-HCW transmission, environmental contamination, and community-acquired disease play important roles. However, exposure risk stratification is likely to take on renewed importance as containment is achieved. Our study highlights the graded risk associated with varying exposure levels in the healthcare setting, which has important implications for workforce return. Our findings lend support to the CDC’s decision to include exposures formerly classified as medium risk in the category to be considered for enhanced postexposure monitoring and work restrictions and highlights the need for further research.
